# Growth Profiles and Temporal Accumulation of Aniline-Blue-Reactive β-(1→3)-Glucan-Associated Material in *Alcaligenes faecalis* A12C Batch Cultures

**DOI:** 10.3390/polym18212749

**Published:** 2026-11-05

**Authors:** C. J. Martel-Benítez, R. Henríquez-Cabrera, R. Alayón-Afonso, D. Padilla, F. Real, A. Martel-Quintana, J. L. Gómez-Pinchetti, C. Almeida, P. Santiago-Díaz, N. Robles-Antón, N. González-Caballero, J. L. Martín-Barrasa

**Affiliations:** 1 University Institute of Animal Health and Food Safety (IUSA-ONE HEALTH) 2-Sanidad Animal de la Acuicultura y Especies Silvestres, Enfermedades Infecciosas y Seguridad Alimentaria, University of Las Palmas de Gran Canaria, 35416 Arucas, Spain; carlosmb1395@gmail.com (C.J.M.-B.); rafael.alayon@ulpgc.es (R.A.-A.); daniel.padilla@ulpgc.es (D.P.); fernando.real@ulpgc.es (F.R.); jmarbars@gobiernodecanarias.org (J.L.M.-B.); 2 Instituto de Investigación Sanitaria de Canarias (IISC), Eukaryotic-Prokaryotic Synergy-Comparative and Translational Medicine Group (Cardiorespiratory Infectious Diseases and Epidemioogy Area), Barranco de la Ballena s/n, 35019 Las Palmas de Gran Canaria, Spain; 3 Fundación Canaria del Instituto de Investigación Sanitaria de Canarias (FIISC), 35010 Las Palmas de Gran Canaria, Spain; 4 Banco Español de Algas, Instituto de Oceanografía y Cambio Global (IOCAG), Universidad de Las Palmas de Gran Canaria, 35214 Telde, Spain; antera.martel@ulpgc.es (A.M.-Q.); jgomez@dbio.ulpgc.es (J.L.G.-P.); carlos.almeida@ulpgc.es (C.A.); paula.santiago@fpct.ulpgc.es (P.S.-D.); robles.noelia@fpct.ulpgc.es (N.R.-A.); nazaret.gonzalez@fpct.ulpgc.es (N.G.-C.); 5 Research Unit, Hospital Universitario de Gran Canaria Dr. Negrín, Fundación del Instituto de Investigación Sanitaria de Canarias (FIISC), 35019 Las Palmas de Gran Canaria, Spain; 6 CIBER de Enfermedades Infecciosas (CIBERINFEC), Instituto de Salud Carlos III, 28029 Madrid, Spain

**Keywords:** *Alcaligenes faecalis*, β-(1→3)-glucan, curdlan, exopolysaccharide, aniline blue, batch culture, growth kinetics, functional biopolymer, triple-helix conformation

## Abstract

Microbial exopolysaccharides (EPSs) are functional biopolymers of growing scientific and industrial interest because of their structural diversity, biocompatibility, and rheological properties. Microbial β-(1→3)-glucans are of particular biological and technological interest, and their production depends strongly on strain characteristics, medium composition, and culture conditions. This study characterized the temporal relationship among bacterial growth, viability, and aniline-blue reactivity in *Alcaligenes faecalis* A12C under batch growth and fermentation conditions. Growth turbidity, viable bacterial concentration, and β-(1→3)-glucan-associated signals were monitored over 96 h using spectrophotometric, microbiological, and microscopic approaches. In growth culture medium (GCM), OD600 increased after 24–30 h and reached approximately 2.4 at 72 h, coinciding with the highest viable count (2.44 × 10^21^ CFU/mL). Aniline-blue coloration became clearly detectable after 30 h and persisted through 96 h, when microscopy showed blue staining associated with bacterial cells. In fermentation culture medium (FCM), OD600 increased progressively to approximately 2.8 at 96 h, whereas viable counts followed a distinct temporal profile. Aniline-blue reactivity changed during the first 24 h and remained close to an A610 of approximately 1.0 thereafter. These findings indicate distinct temporal patterns among culture turbidity, viability, and aniline-blue reactivity and provide a framework for subsequent structural characterization of the detected material.

## 1. Introduction

In recent years, microbial exopolysaccharides (EPSs) have emerged as functional biopolymers with broad applications across the food, pharmaceutical, biomedical, and environmental technology sectors [1,2]. Bacterial EPSs offer exceptional structural diversity, non-toxicity, high biocompatibility, and unique rheological and gelling properties that synthetic polymers cannot easily replicate [3,4]. Among these extracellular polymers, β-glucans represent a heterogeneous group of homopolysaccharides composed of D-glucose units linked by glycosidic bonds, whose physicochemical characteristics and biological activities are dictated by their structural linkage patterns, molecular weights, and solution conformations [5,6]. Extracellular β-(1→3)-D-glucans are particularly valuable functional polymers due to their distinctive conformational features, notably their ability to form stable single- or triple-helical structures in aqueous environments [7,8]. These structural conformations govern both their hydrogel-forming capabilities and their specific binding affinities to pattern-recognition receptors on immune cells, such as Dectin-1 and Toll-like receptors, conferring notable immunomodulatory, anti-inflammatory, antioxidant, and wound-healing bioactivities [9,10,11,12].

Curdlan is an unbranched, linear homopolysaccharide consisting exclusively of β-(1→3)-linked D-glucose residues, originally described in agrobacteria and alcaligenes species [13]. One of its most distinct industrial properties is its capacity to form thermoreversible or irreversible hydrogels upon heating, rendering it an attractive gelling agent, stabilizer, and texture modifier in food systems, as well as a matrix for drug delivery and tissue engineering [14,15]. Curdlan and curdlan-like exopolysaccharides have been reported in bacterial species such as *Alcaligenes faecalis*, *Agrobacterium* spp., *Paenibacillus* spp., and *Rhizobium radiobacter* [14,15,16,17,18,19,20]. In addition, linear β-(1→3)-glucans are synthesized by microalgae (such as paramylon in *Euglena* spp.) and yeasts (such as zymosan in *Saccharomyces cerevisiae*) (Table 1) [21,22,23,24].

To detect and monitor β-(1→3)-glucan production during microbial cultivation, the triphenylmethane dye aniline blue (water-soluble aniline blue/fluorochrome) has been widely utilized as a presumptive screening reagent [24]. Aniline blue specifically interacts with linear β-(1→3)-D-glucan chain linkages, inducing a characteristic colorimetric absorbance shift (measured spectrophotometrically at 610 nm) and specific fluorescence emission [25,26,27]. However, while aniline-blue reactivity provides a rapid operational tool for monitoring polymer appearance in culture media, it remains a presumptive indicator and does not replace definitive chemical characterization.

In previous research by our group, the wild-type strain *Alcaligenes faecalis* A12C—originally isolated from marine fish gills—demonstrated significant immunomodulatory properties and safety in preclinical animal models [18]. Long-term oral administration of strain A12C significantly improved host survival in Sprague Dawley rats subjected to polymicrobial sepsis induced by cecal ligation and puncture (CLP), a protective effect associated with reduced pathogen systemic dissemination and increased fecal accumulation of curdlan-like material [18]. However, those in vivo studies did not characterize the temporal kinetics of exopolysaccharide secretion by A12C under controlled batch fermentation conditions, nor did they determine the operational relationship between cell viability, culture turbidity, and polymer accumulation.

Accordingly, the primary purpose of this study was to evaluate the growth kinetics and temporal dynamics of aniline-blue-reactive β-(1→3)-glucan-associated exopolysaccharide accumulation in *A. faecalis* A12C batch cultures across different media compositions (growth culture medium vs. fermentation culture medium). This research establishes the essential bioprocessing baseline required for bioreactor optimization and downstream polymer purification.

## 2. Materials and Methods

### 2.1. Alcaligenes faecalis A12C Strain

The bacterial strain *Alcaligenes faecalis* subsp. *faecalis* A12C (A12C) was isolated from the gills of *Argyrosomus regius* at the University Institute of Animal Health and Food Safety (IUSA), University of Las Palmas de Gran Canaria, Spain [18]. The strain has been deposited in the Spanish Type Culture Collection (CECT, Valencia, Spain) under accession CECT 31431 (Patent OEPM P202631159). The present manuscript represents an in vitro microbiological and bioprocess characterization of strain A12C in liquid batch cultures; no animal experimentation, blood chemistry, or hematology was performed in this study.

The strain was maintained at −80 °C in Brain Heart Infusion broth supplemented with 20% (*v*/*v*) glycerol, as previously described [18]. Fresh cultures were obtained from Trypticase Soy Agar supplemented with 5% sheep blood (Becton Dickinson, Franklin Lakes, NJ, USA) and incubated aerobically at 37 °C before use.

### 2.2. Growth Culture Medium and Growth Curve

One liter of growth culture medium (GCM) was prepared using a formulation modified from those previously described by Zhang et al. [28] and Jiang et al. [29] by adding MgSO_4_·7H_2_O at 0.49 g/L and (NH_4_)_2_HPO_4_ at 1.6 g/L to improve pH stability during bacterial growth. The original formulation described in the literature is referred to as reference GCM in Appendix A.1, whereas the modified buffered formulation was the medium actually used in the present experiments and is referred to as GCM throughout the manuscript. The final composition of the medium is shown in Appendix A.1. Culture pH was measured at 0, 2, 4, 6, 24, 30, and 48 h using a portable pH meter calibrated before each use. The pH was monitored and remained stable throughout the culture. Unless otherwise indicated, analytical measurements were performed in duplicate.

A preliminary single-batch growth-curve experiment was performed exclusively in GCM to characterize the temporal growth profile of *A. faecalis* A12C and to select the growth stage used for subsequent inoculation of FCM. This experiment was independent of the subsequent comparison of viable bacterial concentrations in GCM and FCM. OD600 was measured in triplicate at each sampling time from the same batch culture.

Fresh colonies of *A. faecalis* A12C were suspended in 6 mL of sterile 0.9% saline solution and adjusted to a 0.5 McFarland standard. This standardized 0.5 McFarland suspension was used to prepare the GCM inoculum. The bacterial suspension was centrifuged at 8000× *g* for 30 min at 4 °C following an adaptation of previously described procedures for curdlan production [17]. The resulting pellet was resuspended in GCM. Submerged batch cultivation was carried out in 250 mL baffled Erlenmeyer flasks containing 50 mL of medium, corresponding to a 20% working volume. Cultures were incubated aerobically at 37 °C under continuous agitation at approximately 100 rpm. In addition, brief CO_2_ pulses were manually applied for 1 min every hour solely to promote culture movement. CO_2_ was not used to establish a controlled atmosphere or as a controlled fermentation parameter. Gas flow rate and dissolved oxygen were not monitored.

**Figure 1:**
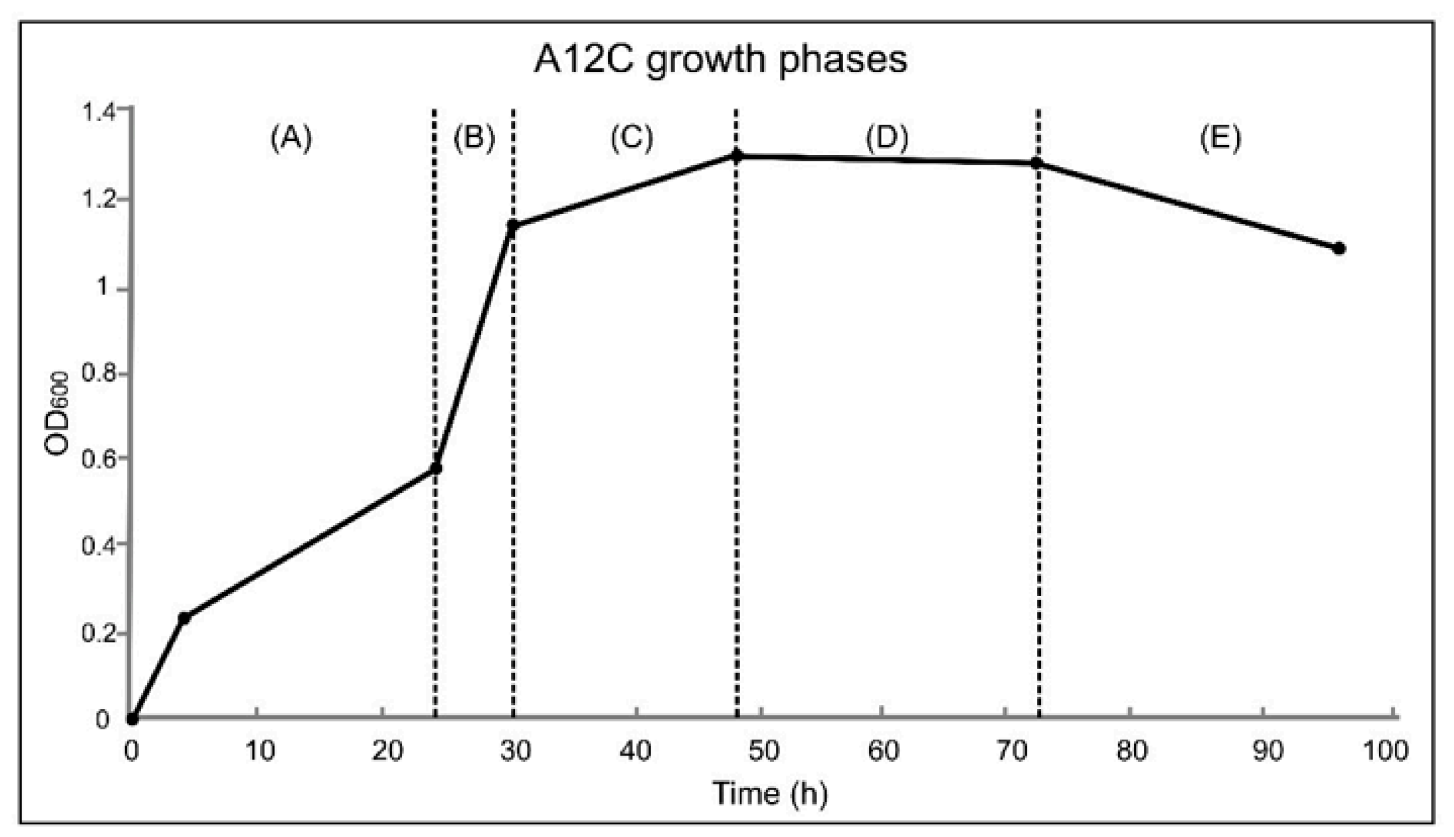
Growth kinetics of *Alcaligenes faecalis* A12C in growth culture medium (GCM) during 96 h of incubation. Bacterial growth was monitored by optical density at 600 nm (OD600). The operationally defined growth phases are indicated as follows: (**A**) lag phase, (**B**) exponential growth phase, (**C**) deceleration phase, (**D**) stationary phase, and (**E**) decline phase. The curve represents the mean of three technical OD600 measurements obtained from a single batch culture at each sampling time; the corresponding standard deviations are reported in Appendix A.2.

Aliquots were collected at 0, 2, 4, 6, 24, 30, 48, 72, and 96 h after inoculation. Bacterial growth was monitored by measuring optical density at 600 nm (OD600) (Figure 1) using a UV-1900i UV–Vis spectrophotometer (Shimadzu, Duisburg, Germany). Optical density was defined as

OD_600_ = −log_10_(I/I_0_) (1)

where (I_0_) is the incident light intensity, and (I) is the transmitted light intensity at 600 nm [30]. OD600 values were used as a dimensionless measure of culture turbidity and were not converted directly to viable-cell concentration. In parallel, 50 µL of each aliquot was used for viable bacterial counts. Three technical OD600 measurements were obtained from the same culture at each sampling time.

### 2.3. Microbiological Counts (CFUs)

Viable bacterial concentrations were determined by serial ten-fold dilution and colony counting. For each sampling time, 50 µL of culture was serially diluted ten-fold in sterile 0.9% saline solution using 96-well plates [18]. From the appropriate dilutions, 25 µL was plated onto MacConkey agar plates (Becton Dickinson) and incubated at 37 °C for 24 h. Colonies of *A. faecalis* A12C were counted after 18–24 h to determine viable bacterial concentration and to verify culture purity.

Viable bacterial concentrations were calculated according to

(2) $$\frac{\mathrm{CFU}}{\mathrm{mL}}=\frac{N}{\left(V*d\right)}$$

where CFU is colony-forming unit, (N) is the number of colonies counted, (V) is the plated volume in mL, and (d) is the corresponding dilution [31]. Because 25 µL (0.025 mL) was plated in the present study, the equation was equivalent to

(3) $$\frac{\mathrm{CFU}}{\mathrm{mL}}=N*40*\frac{1}{d}$$

CFU/mL values were assigned to the corresponding culture sampling time and represent viable, cultivable A12C cells present in the sampled aliquot. The subsequent 18–24 h incubation on solid medium was used only to allow colony development and did not represent additional fermentation time. CFU counts were not interpreted as a direct measure of polysaccharide-producing cells.

### 2.4. Presumptive Detection of β-(1→3)-Glucan on Aniline Blue Agar

The polysaccharide investigated in this study was a curdlan-type β-(1→3)-D-glucan-associated material. Curdlan is a linear homopolysaccharide composed of D-glucose residues linked through β-(1→3) glycosidic bonds [32]. However, because no structural identification was performed in the present study, the detected material was operationally considered an aniline-blue-reactive β-(1→3)-glucan-associated polysaccharide rather than chemically confirmed curdlan.

**Figure 2:**
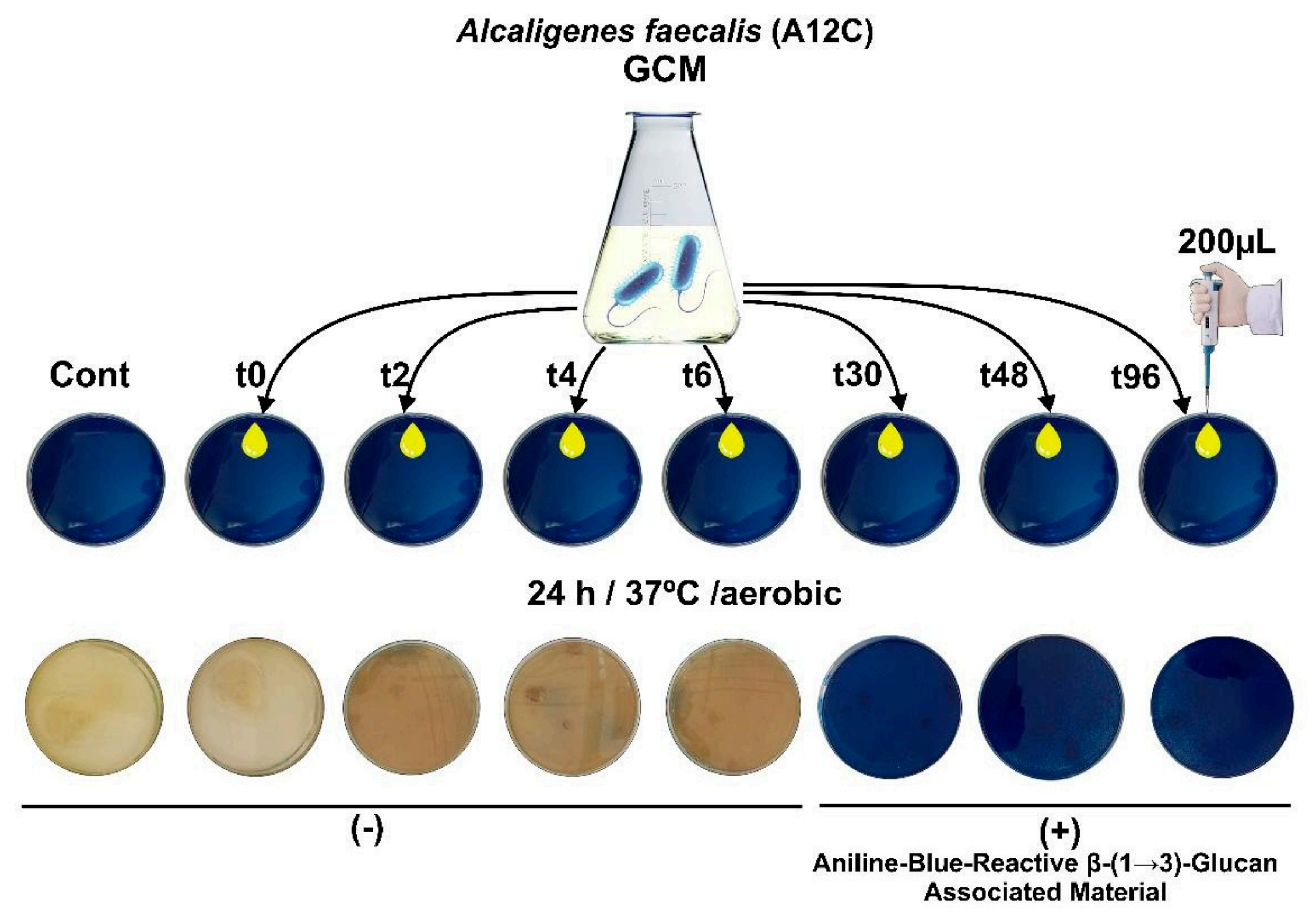
Presumptive detection of aniline-blue-reactive β-(1→3)-glucan-associated material produced by *Alcaligenes faecalis* A12C. Representative aniline-blue agar plates corresponding to culture aliquots collected at selected sampling times (t0, t2, t4, t6, t30, t48, and t96 h) and incubated aerobically at 37 °C for 24 h. No clear blue coloration was observed during the initial incubation period, whereas distinct blue coloration became evident from approximately 30 h onward and persisted through 96 h.

Aniline blue (VWR, BDH Chemicals, Radnor, PA, USA.) was prepared at a concentration of 200 µg/mL in 0.1 M citrate buffer (pH 7.0). Aniline blue agar was prepared in accordance with the previously described protocol [18]. The formulation contained sucrose (20 g/L), yeast extract (5 g/L), peptone (5 g/L), aniline blue (0.05 g/L), and agar (20 g/L), and the final pH was adjusted to 7.2 [33]. The basal medium was autoclaved at 121 °C for 15 min. Aniline blue was incorporated into the medium for the presumptive detection of β-(1→3)-glucan-producing bacteria. After solidification, bacterial samples were inoculated onto the plates and incubated aerobically at 37 °C for 24 h. Development of blue coloration was interpreted as a presumptive positive signal for β-(1→3)-glucan-associated material. Representative aniline-blue agar plates corresponding to selected culture sampling times are shown in Figure 2.

### 2.5. Fermentation Culture Medium

The fermentation culture medium (FCM) was prepared according to the composition shown in Appendix A.1 and based on previously described media for curdlan production [28,29,33,34,35]. FCM contained maltose (70.0 g/L), urea (0.64 g/L), (NH_4_)_2_SO_4_ (1.0 g/L), KH_2_PO_4_ (1.0 g/L), MgSO_4_·7H_2_O (0.5 g/L), and NaCl (1.0 g/L). The trace-element solution was prepared separately according to the composition shown in Appendix A.1 and added to FCM at 10 mL/L. A 24 h culture of *A. faecalis* A12C grown in GCM was used to inoculate FCM at 10% (*v*/*v*). Submerged batch cultures were performed in 250 mL baffled Erlenmeyer flasks containing 50 mL of FCM. Cultures were incubated aerobically at 37 °C under continuous agitation at approximately 100 rpm, with additional manually applied CO_2_ pulses for 1 min every hour to promote culture movement, as described for GCM.

Aliquots were collected at 0, 2, 4, 6, 24, 30, 48, 72, and 96 h after inoculation. At each sampling time, bacterial growth, viable bacterial concentration, and aniline-blue reactivity were evaluated. The pH was monitored and remained stable throughout the culture.

### 2.6. Alkaline Extraction and Aniline Blue Colorimetric Assay

For the assessment of aniline-blue-reactive material, culture aliquots collected at 0, 2, 4, 6, 24, 30, 48, 72, and 96 h were centrifuged at 4000× *g* for 15 min at 4 °C, following an adaptation of previously described procedures for curdlan fermentation and recovery [35]. The resulting pellets were resuspended in 0.1 M NaOH and incubated at 60 °C for 30 min. After alkaline treatment, samples were centrifuged again, and the resulting supernatant was recovered for analysis.

The recovered alkaline supernatant was subsequently mixed with an equal volume of aniline blue solution (200 µg/mL in 0.1 M citrate buffer, pH 7.0) (1:1, *v*/*v*) and incubated for 30 min at room temperature in darkness. Absorbance was measured at 610 nm (A610) using 1 mL cuvettes in a UV-1900i UV–Vis spectrophotometer (Shimadzu, Duisburg, Germany). Determinations were performed in duplicate. Sterile medium blanks subjected to the same extraction and staining procedure were used for baseline correction, and commercial curdlan from *Alcaligenes faecalis* (Sigma-Aldrich, Burlington, MA, USA) was included as a positive control. Aniline-blue reactivity was considered an indirect estimate of β-(1→3)-glucan-associated material and was not interpreted by itself as structural confirmation of curdlan [27].

### 2.7. Optical Microscopy

For microscopic examination, aniline blue (VWR, BDH Chemicals, Radnor, PA, USA) was prepared at 200 µg/mL in 0.1 M citrate buffer (pH 7.0). Uncentrifuged samples collected after 96 h of incubation were mixed with an equal volume of aniline blue solution (1:1, *v*/*v*) and examined by optical microscopy. Microscopic evaluation was performed to assess the distribution of aniline blue staining in relation to bacterial cells. This qualitative examination was not designed to determine the subcellular localization of the staining or to distinguish extracellular/cell-associated material from possible intracellular staining.

### 2.8. Data Analysis

Experimental determinations were performed in duplicate. For the preliminary GCM growth-curve experiment, OD600 was measured in triplicate at each sampling time from a single batch culture. These triplicate measurements represent technical replicates, and the results are reported as mean ± standard deviation (SD) in Appendix A.2. Data were summarized descriptively and graphically represented using Microsoft Excel (Microsoft Corporation, Redmond, WA, USA). Optical density values are reported as dimensionless OD600 measurements; viable bacterial concentrations are expressed as CFU/mL, and aniline-blue reactivity is reported as absorbance measured at 610 nm. OD600, CFU/mL, and A610 were treated as independent analytical measurements. No calibration curve relating OD600 to CFU/mL was generated; therefore, OD610 was not interpreted as a quantitative measure of cell viability. Their temporal values are provided in Appendix A.3 for descriptive comparison.

No inferential statistical analyses were performed.

### 2.9. Use of Generative Artificial Intelligence

While preparing this manuscript, the authors used ChatGPT (OpenAI, GPT-5.6 Sol) to help refine the language, organize, and draft the manuscript text based on the scientific content they provided in order to adapt it to the journal’s format. The tool was not used to generate or modify experimental data, perform statistical analyses, or independently determine the scientific conclusions of the study.

## 3. Results

### 3.1. Growth Kinetics of Alcaligenes faecalis A12C in GCM

The growth of *Alcaligenes faecalis* A12C in growth culture medium (GCM) was monitored for 96 h by measuring optical density at 600 nm (OD600). The growth profile showed five operationally defined phases: lag, exponential growth, deceleration, stationary, and decline (Figure 1). OD600 remained relatively stable during the first 24–30 h of incubation and subsequently increased markedly, reaching its highest observed value of approximately 1.272 at 72 h. Thereafter, OD600 remained comparatively stable or decreased slightly by 96 h. Mean OD600 values and the corresponding standard deviations of the triplicate technical measurements are provided in Appendix A.2.

These results indicate that the modified GCM supported sustained growth of *A. faecalis* A12C, with the greatest increase in culture turbidity occurring between 30 and 72 h.

### 3.2. Presumptive Detection of β-(1→3)-Glucan-Associated Material

Aniline-blue reactivity was evaluated as a presumptive indicator of β-(1→3)-glucan-associated material produced during bacterial growth. Blue coloration on aniline-blue agar was first clearly observed in aliquots collected after 30 h of incubation and remained detectable until 96 h (Figure 2).

**Figure 3:**
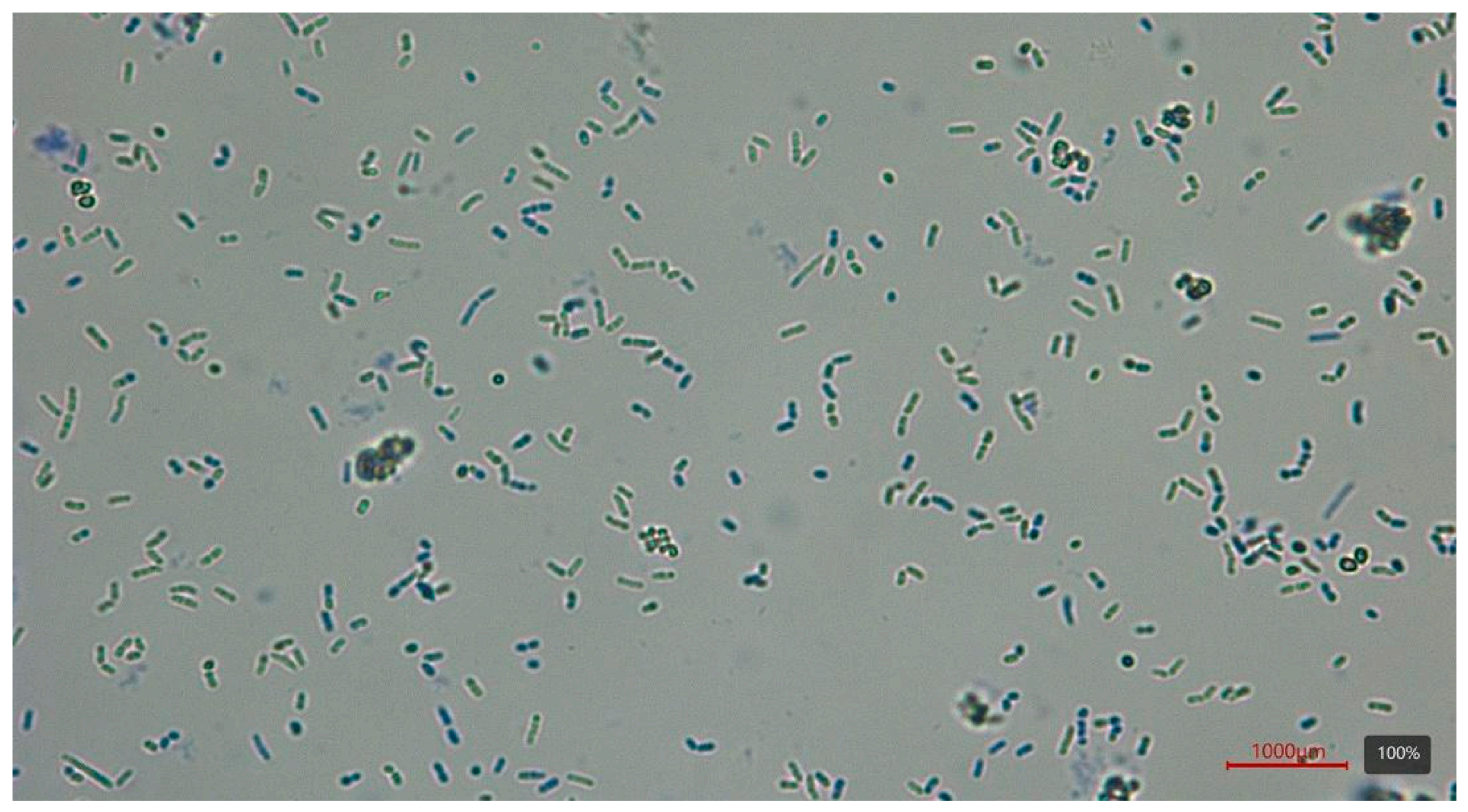
Presumptive detection of β-(1→3)-glucan-associated material produced by *Alcaligenes faecalis* A12C. Aniline-blue reactivity observed during bacterial growth; representative optical microscopy image of a 96 h culture after staining with aniline blue. The image shows the spatial association of blue-stained material with bacterial cells but does not allow discrimination between extracellular, cell-associated, or intracellular localization.

At 96 h, optical microscopy of uncentrifuged samples mixed with aniline blue showed blue staining associated with bacterial cells (Figure 3). However, conventional optical microscopy did not allow discrimination between extracellular or cell-associated staining and possible intracellular localization. These observations are consistent with the presence of β-(1→3)-glucan-containing material associated with *A. faecalis* A12C. However, aniline-blue staining alone does not establish the chemical identity of the polymer as curdlan.

### 3.3. Viable Bacterial Counts in GCM and FCM

**Figure 4:**
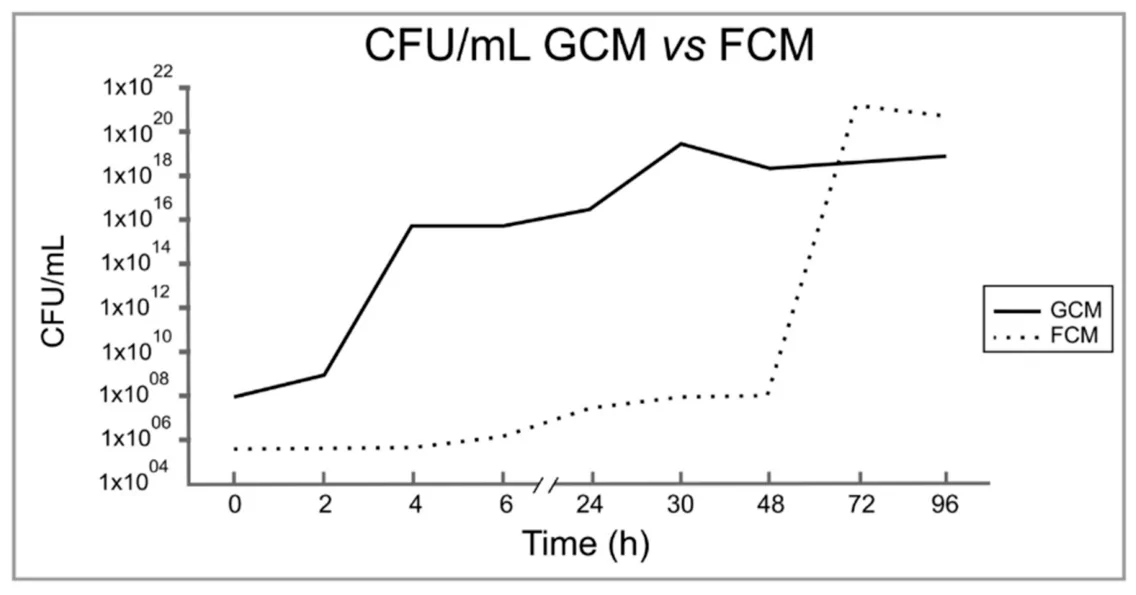
Viable bacterial concentrations of *Alcaligenes faecalis* A12C in growth culture medium (GCM) and fermentation culture medium (FCM) during 96 h of incubation. Results are expressed as CFU/mL.

Viable bacterial concentrations showed different temporal profiles in GCM and fermentation culture medium (FCM) (Figure 4). In GCM, viable counts remained relatively stable during the first 48 h and subsequently increased markedly, reaching their highest observed concentration at 72 h. At this time point, the bacterial concentration reached approximately 2.44 × 10^21^ CFU/mL. A subsequent decrease to approximately 1 × 10^21^ CFU/mL was observed at 96 h.

In FCM, viable bacterial concentrations remained around 10^8^ CFU/mL during the first 24 h and subsequently increased, reaching approximately 5 × 10^19^ CFU/mL during the following growth period. Viable counts decreased to approximately 10^18^ CFU/mL at 48 h and then increased gradually, reaching approximately 1 × 10^19^ CFU/mL at 96 h.

Thus, both media supported viable growth of *A. faecalis* A12C, although the magnitude and temporal pattern of viable bacterial concentrations differed between GCM and FCM.

### 3.4. Temporal Profiles of OD600 and Viable Bacterial Counts in GCM

**Figure 5:**
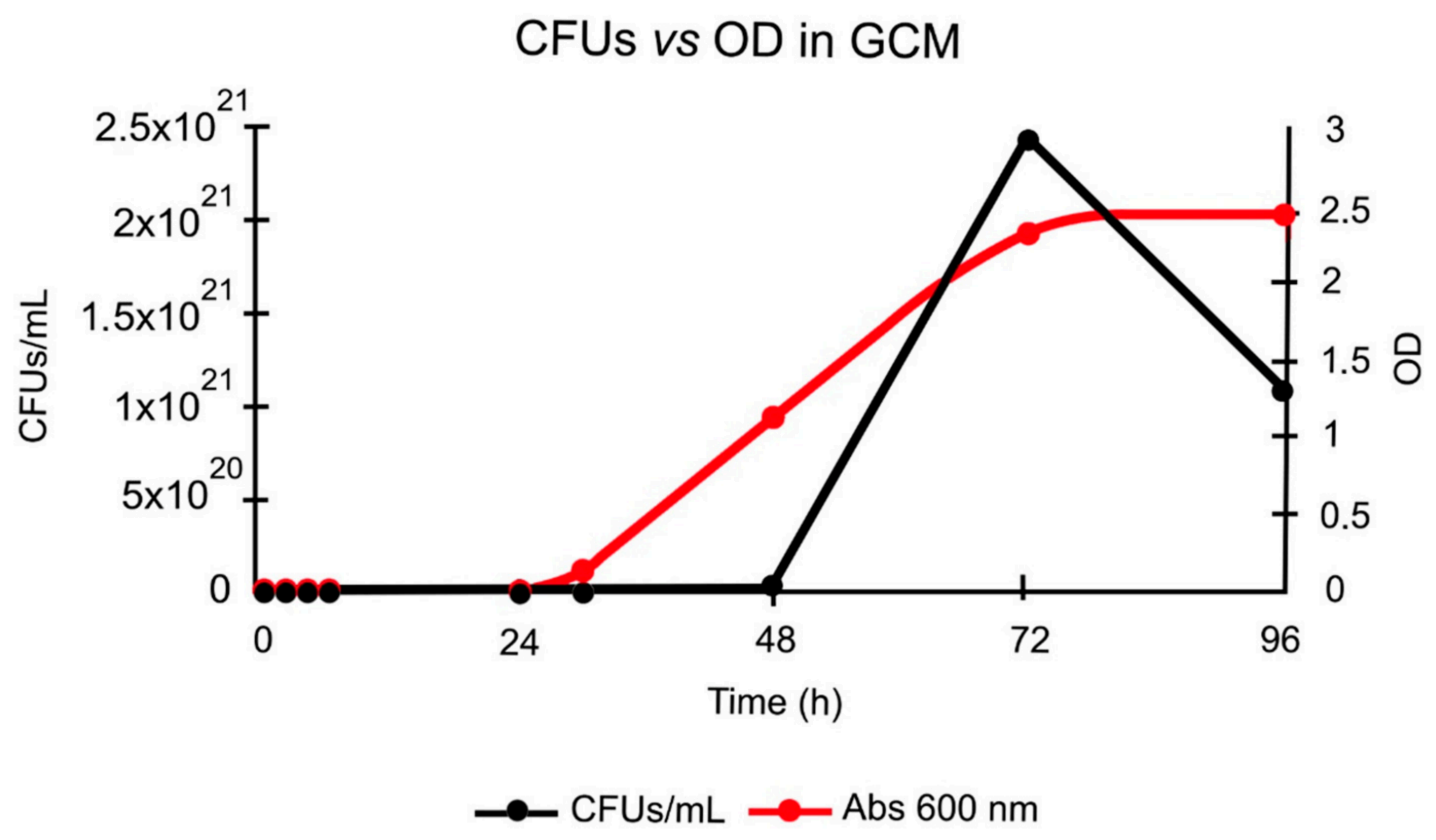
Temporal profiles of optical density at 600 nm (OD600) and viable bacterial concentration of *Alcaligenes faecalis* A12C in growth culture medium (GCM) during 96 h of incubation. Viable bacterial concentrations are expressed as CFU/mL. The parameters are presented together for descriptive temporal comparison and should not be interpreted as quantitatively equivalent measurements. Individual values are provided in Appendix A.3.

The temporal profiles of OD600 and viable bacterial concentration in GCM are shown in Figure 5. During the first 30 h of incubation, OD600 remained relatively stable, while viable bacterial concentrations showed limited variation during the first 48 h. Thereafter, both parameters increased markedly, although their subsequent temporal behavior differed.

At 72 h, OD600 reached approximately 2.3, while the viable bacterial concentration reached 2.44 × 10^21^ CFU/mL, its highest observed value. At 96 h, viable bacterial concentration decreased to 1.08 × 10^21^ CFU/mL, corresponding to an approximately 56% reduction, whereas OD600 remained high and increased slightly to approximately 2.4.

Therefore, OD600 and viable bacterial counts showed a similar temporal increase during the active growth period but diverged during the later stage of incubation. The persistence of high OD600 values despite the reduction in viable counts indicates that culture turbidity did not exclusively reflect viable bacterial concentration at the end of the experiment. OD600 may reflect contributions from viable and non-viable cells, cellular aggregates, and extracellular material; therefore, it was not interpreted as a direct surrogate for viable bacterial concentration. The individual OD600 and CFU/mL values obtained at each sampling time are provided in Appendix A.3.

### 3.5. Growth, Viable Counts, and Aniline-Blue Reactivity in FCM

**Figure 6:**
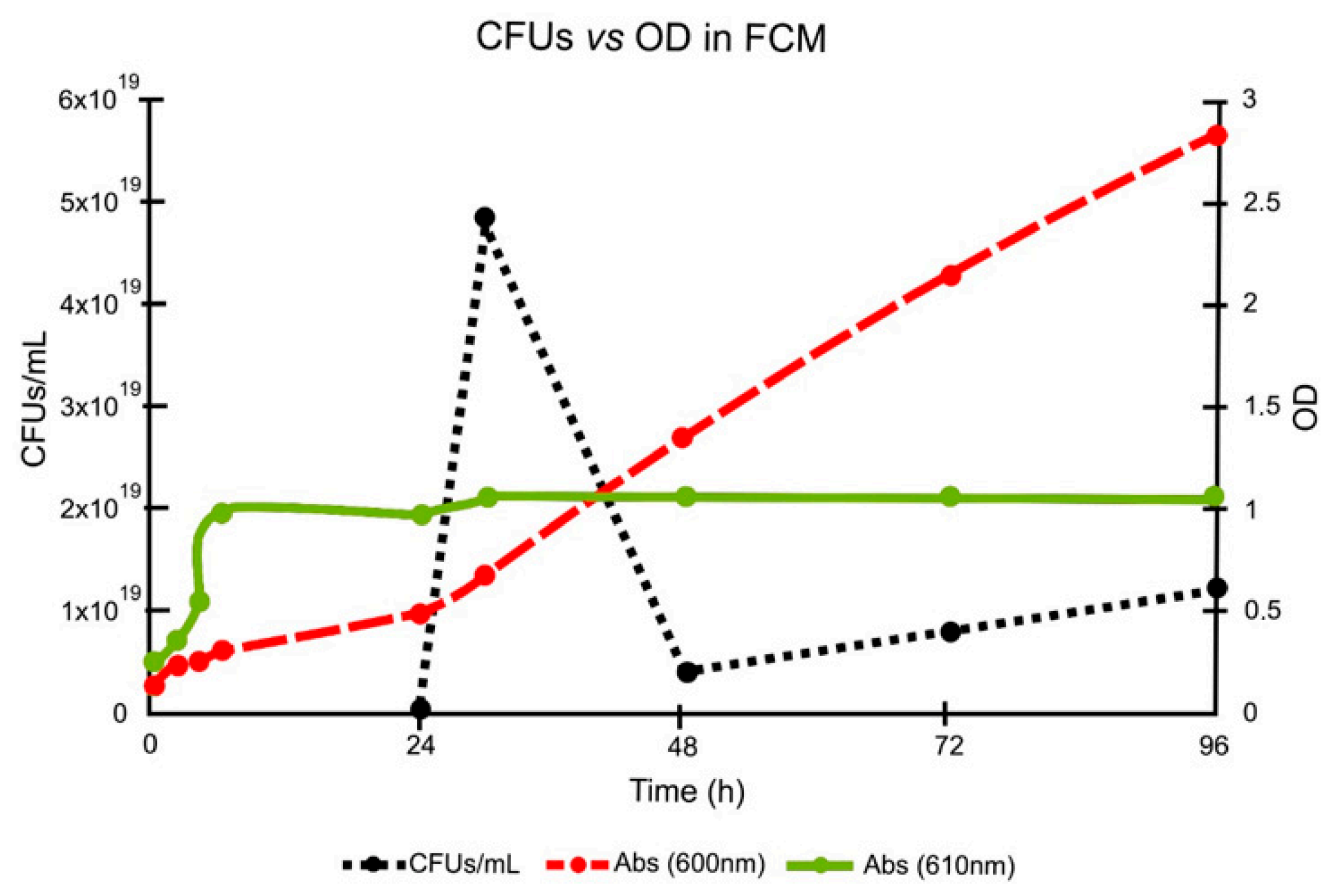
Temporal profiles of optical density at 600 nm (OD600), viable bacterial concentration, and aniline-blue reactivity at 610 nm (A610) in *Alcaligenes faecalis* A12C cultured in fermentation culture medium (FCM) for 96 h. Viable bacterial concentrations are expressed as CFU/mL. These parameters are presented together for descriptive temporal comparison and should not be interpreted as quantitatively equivalent measurements. Individual values are provided in Appendix A.3.

In FCM, OD600 increased progressively throughout the incubation period, particularly after 24 h, and reached its highest observed value of approximately 2.8 at 96 h (Figure 6). In contrast, viable bacterial concentration increased from 1.20 × 10^8^ CFU/mL at 0 h to 4.16 × 10^16^ CFU/mL at 24 h and reached its highest observed value of 4.80 × 10^19^ CFU/mL at 30 h. Viable counts subsequently decreased to 4.00 × 10^18^ CFU/mL at 48 h and then increased gradually to 1.20 × 10^19^ CFU/mL at 96 h.

Aniline-blue reactivity was already detectable during incubation. Absorbance at 610 nm (A610) increased during the first 6 h and subsequently remained at approximately 0.9–1.0 until 24 h. Thereafter, A610 remained comparatively stable at approximately 1.0 through 96 h.

The temporal profiles of OD600, viable bacterial counts, and A610 differed during fermentation. In particular, the continued increase in OD600 during the later stages of incubation was not accompanied by a proportional increase in viable bacterial concentration. Similarly, the comparatively stable A610 values observed after approximately 24 h occurred despite subsequent fluctuations in viable bacterial concentration. This pattern indicates that aniline-blue reactivity and viable bacterial concentration followed distinct temporal profiles; however, A610 was not interpreted as a direct measure of polymer synthesis, polymer concentration, or cell viability. The individual OD600, CFU/mL, and A610 values obtained at each sampling time are provided in Appendix A.3.

## 4. Discussion

The present study characterized the temporal relationship among bacterial growth, cell viability, and the development of an aniline-blue-reactive signal compatible with β-(1→3)-glucan-associated material in *Alcaligenes faecalis* A12C under batch culture conditions. GCM and FCM produced distinct temporal profiles of viable bacterial concentration, optical density, and aniline-blue reactivity. These findings indicate that bacterial proliferation and the development of the aniline-blue-reactive signal followed distinct temporal patterns that varied according to culture medium. Importantly, aniline-blue reactivity was used as a presumptive indicator of β-(1→3)-glucan-associated material and should not be interpreted as chemical identification of curdlan. Nevertheless, the temporal monitoring performed in this study provides information on the culture stages in which this polysaccharide-associated signal develops and persists.

Aniline blue has been widely used as a screening reagent for curdlan-type and other β-(1→3)-glucans because of its interaction with these polysaccharide structures [29]. Previous studies have also described spectroscopic changes in aniline blue upon interaction with β-(1→3)-glucans, supporting its use as a presumptive analytical probe [26,27]. In the present study, blue coloration became clearly detectable after approximately 30 h of incubation and remained detectable through the later stages of culture. The development of A610 reactivity together with the microscopic association of blue-stained material with bacterial cells provides operational evidence for the presence of aniline-blue-reactive β-(1→3)-glucan-associated material but does not establish its extracellular localization or chemical identity. This temporal pattern coincided with the transition from the initial growth period toward later phases of incubation. Previous studies have shown that curdlan production in confirmed bacterial producers is strongly influenced by medium composition, carbon and nitrogen availability, and fermentation conditions and may be favored when continued biomass formation becomes restricted while an adequate carbon source remains available [24,36,37]. The temporal pattern observed in A12C is therefore compatible with production behavior previously described for curdlan-producing bacteria. However, because aniline blue is not chemically specific for curdlan, the present findings should be interpreted as evidence of an aniline-blue-reactive β-(1→3)-glucan-associated material rather than definitive identification of the polymer.

The relationship between OD600 and viable bacterial counts further supports this interpretation. In the experiment in which both parameters were monitored simultaneously in GCM, OD600 and viable bacterial concentration increased during the active growth period. At 72 h, viable bacterial concentration reached its highest observed value (2.44 × 10^21^ CFU/mL), whereas OD600 was approximately 2.3. By 96 h, viable bacterial concentration had decreased by approximately 56%, while OD600 remained high at approximately 2.4. A similar divergence was observed in FCM, where OD600 continued to increase without a proportional increase in viable bacterial concentration. Optical density reflects total culture turbidity rather than viable-cell number alone. Consequently, non-viable cells, cellular aggregates, and extracellular or cell-associated material may contribute to OD600 during prolonged incubation. Similar divergence between optical density and viable-cell measurements has been reported in microbial cultures producing extracellular polymers [28,29,34]. In microorganisms producing extracellular polysaccharides, polymer accumulation may also modify the physical characteristics of the culture medium and contribute to turbidity or viscosity [36,37]. Although the relative contribution of these components cannot be determined from the present experiments, the observed divergence indicates that OD600 alone is not an adequate surrogate for viable bacterial concentration throughout the entire culture period. No OD600–CFU/mL calibration curve was established in the present study, and no quantitative equivalence between these parameters was assumed. Accordingly, OD600 and viable bacterial counts were interpreted independently and compared only descriptively according to their temporal profiles.

In FCM, aniline-blue reactivity also followed a temporal pattern that differed from that of viable bacterial counts. The signal changed during the first 24 h and remained comparatively stable from approximately 24 h onward, whereas viable bacterial concentrations showed a more variable profile. This divergence indicates that the intensity of the aniline-blue-reactive signal at a given sampling point does not necessarily parallel the number of viable cells present at the same time. No quantitative relationship between A610 and viable bacterial concentration was established in the present study; consequently, aniline-blue reactivity should be interpreted as an independent indicator of β-(1→3)-glucan-associated material rather than as a marker of cell viability. The persistence of this signal during the later stages of incubation may reflect the continued presence and/or accumulation of β-(1→3)-glucan-associated material generated during previous stages of culture. Similar production patterns have been described in established curdlan-producing bacteria, in which polysaccharide formation or persistence may extend beyond the period of most active cellular proliferation [22,36]. Nevertheless, the present data do not allow the relative contribution of newly synthesized material and previously accumulated polymer to be distinguished.

The observed temporal behavior is consistent with the initial hypothesis that the aniline-blue-reactive signal would become more evident as cultures progressed from active bacterial growth toward later stages of incubation. However, this relationship should be interpreted descriptively. The study did not directly measure residual carbon or nitrogen concentrations and therefore cannot determine which nutritional change, if any, triggered the observed changes in aniline-blue reactivity. In particular, nitrogen limitation cannot be inferred solely from the temporal pattern. Direct monitoring of substrate consumption and nutrient availability would be required to establish the metabolic conditions associated with the development of aniline-blue reactivity in A12C.

A relevant contribution of the present study is the simultaneous temporal assessment of culture turbidity, bacterial viability, and aniline-blue reactivity rather than evaluation of a single fermentation endpoint. This approach allowed the development of the β-(1→3)-glucan-associated signal to be examined in relation to changes in bacterial growth and identified culture stages that may be particularly relevant for subsequent polymer recovery and characterization. The comparison between GCM and FCM further showed that conditions supporting bacterial proliferation do not necessarily produce parallel changes in aniline-blue reactivity, highlighting the importance of evaluating biomass formation and polysaccharide-associated signals independently during microbial fermentation.

Several limitations should be considered. The study was designed as a descriptive temporal characterization; most analytical determinations were performed in duplicate; and the preliminary GCM growth-curve experiment included triplicate technical OD600 measurements from a single batch culture. No inferential statistical analyses were conducted. Accordingly, the observed differences and temporal patterns should not be interpreted as statistically demonstrated effects. In addition, aniline-blue reactivity provides presumptive evidence of β-(1→3)-glucan-associated material but does not establish polymer identity, purity, molecular structure, or absolute production yield. The optical microscopy analysis was qualitative and did not permit determination of whether aniline-blue-reactive material was localized exclusively in an extracellular layer, associated with the cell surface, or present intracellularly. Furthermore, the contribution of extracellular material to OD600 could not be distinguished from that of bacterial biomass, and nutrient concentrations were not monitored throughout fermentation.

### Future Perspectives

Future studies should combine the temporal framework established here with isolation and purification of the aniline-blue-reactive material followed by independent structural characterization. Techniques such as one- and two-dimensional nuclear magnetic resonance (NMR) spectroscopy and Fourier-transform infrared (FTIR) spectroscopy could be used to determine glycosidic linkage patterns and support definitive polymer identification. Molecular-weight distribution and macromolecular characteristics could subsequently be investigated using size-exclusion chromatography coupled with multi-angle light scattering (SEC-MALS), together with complementary approaches for evaluating polymer conformation.

From a bioprocess perspective, controlled stirred-tank cultivation would allow the effects of dissolved oxygen, pH, agitation, and carbon-to-nitrogen ratio on biomass formation and aniline-blue-reactive material production to be evaluated systematically. Once the polymer has been purified and structurally identified, functional studies could then assess its biological activity and potential receptor-mediated interactions, including β-glucan-responsive pathways where appropriate.

## 5. Conclusions

*Alcaligenes faecalis* A12C developed an aniline-blue-reactive signal compatible with β-(1→3)-glucan-associated material during submerged batch cultivation under the conditions evaluated. The appearance and persistence of this signal followed a temporal pattern distinct from viable bacterial concentration and optical density, particularly under fermentation conditions. These findings indicate that bacterial proliferation and the development of aniline-blue-reactive polysaccharide-associated material do not necessarily follow parallel temporal profiles. In GCM, the highest viable bacterial concentration was observed at 72 h (2.44 × 10^21^ CFU/mL), whereas in FCM, aniline-blue reactivity became comparatively stable from approximately 24 h onward despite subsequent changes in viable bacterial concentration.

The present study provides a temporal framework for monitoring β-(1→3)-glucan-associated material in A12C cultures and identifies culture stages of interest for subsequent polymer recovery and characterization. This framework provides an operational basis for subsequent bioprocess optimization and structural characterization. However, aniline-blue reactivity alone cannot establish the chemical identity of the detected material as curdlan, and independent structural and quantitative analyses, including NMR and FTIR-based approaches, are required for definitive polymer identification.

## 6. Patents

A patent application related to the A12C strain and its potential applications has been filed with the application number P202631159 in the Spanish Patent and Trademark Office (OEPM; Oficina Española de Patentes y Marcas).

## Figures and Tables

**Table 1:** Comparative overview of source, structural features, and main biological actions/applications of representative microbial β-glucans and exopolysaccharides.

Polysaccharide	Producing Source/Organism	Glycosidic Linkage & Structural Features	Main Biological Action/Application
Curdlan (target EPS) Braian et al. [16] Mohsin et al. [17] Martel-Benitez et al. [18]	*Alcaligenes faecalis* (and *A. faecalis* ATCC 31749)	Linear β-(1→3)-D-glucan; triple-helix conformation in hydrogels	Innate immune training (Dectin-1 binding), anti-sepsis protection, thermal gelation, texture modifier
Succinoglycan Wu et al. [14]	*Agrobacterium tumefaciens*/ *Rhizobium meliloti*	Octasaccharide repeating units with β-(1→3), β-(1→4), β-(1→6) linkages	Rheological modification, food thickening, enhanced oil recovery, bio-flocculation
Exopolysaccharide Glucan Rafigh et al. [19]	*Paenibacillus polymyxa*	Branched β-(1→3), β-(1→6)-D-glucan complex	Immunomodulation, antioxidant defense, biological control against plant pathogens
Date-Juice Curdlan Salah et al. [20]	*Rhizobium radiobacter* ATCC 6466TM	Linear β-(1→3)-D-glucan homopolymer	Syrup gelation, food film formulation, biopharmaceutical emulsifier
Paramylon Feuzingt et al. [21]	*Euglena gracilis* (*Euglenoid Microalga*)	Unbranched linear insoluble β-(1→3)-D-glucan granules	Macrophage activation, metabolic regulation, wound dressing, anti-hyperlipidemic agent
Yeast β-Glucan/Zymosan Lesage et al. [22] Bozoudi et al. [23]	*Saccharomyces cerevisiae*	β-(1→3)-D-glucan backbone with β-(1→6) side chains	Innate immune stimulation, vaccine adjuvant, dietary prebiotic fiber

## Data Availability

The data supporting the findings of this study are available from the corresponding author upon reasonable request.

## Abbreviations

The following abbreviations are used in this manuscript:

A610	Absorbance at 610 nm
CECT	Spanish Type Culture Collection (Colección Española de Cultivos Tipo)
CFU	Colony-forming unit
EPS	Exopolysaccharide
FCM	Fermentation culture medium
GCM	Growth culture medium
IUSA	Instituto Universitario de Sanidad Animal y Seguridad Alimentaria
NMR	Nuclear magnetic resonance
OD600	Optical density at 600 nm
OEPM	Spanish Patent and Trademark Office (Oficina Española de Patentes y Marcas)
rpm	Revolutions per minute
SD	Standard deviation
SEC-MALS	Size-exclusion chromatography coupled with multi-angle light scattering
FTIR	Fourier-transform infrared spectroscopy
ULPGC	University of Las Palmas de Gran Canaria (Universidad de Las Palmas de Gran Canaria)
UV–Vis	Ultraviolet–visible
*v*/*v*	Volumen/Volumen

## Appendix A

### Appendix A.1

**Table A1:** Composition of the reference growth culture medium (reference GCM), the buffered GCM used experimentally (GCM), the fermentation culture medium (FCM), and the trace-element solution in grams per liter of purified water following the modified protocols of Zhang et al. [28] and Jiang et al. [29].

Reagents	Reference GCM [28,29] (1 L)	GCM (1 L)	FCM (1 L)	Traces Solution (1 L)
Glucose (Sigma-Aldrich, Burlington, MA, USA)	20 g	20 g	—	—
Yeast extract (PanReac AppliChem. Barcelona, Spain)	10 g	10 g	—	—
KH_2_PO_4_ (Sigma-Aldrich, Burlington, MA, USA)	2.6 g	2.6 g	1 g	—
MgSO_4_·7H_2_O (Sigma-Aldrich, Burlington, MA, USA)	0.49 g	0.49 g	0.5 g	—
(NH_4_)_2_HPO_4_ (Sigma-Aldrich, Burlington, MA, USA)	—	1.6 g	1.5 g	—
Maltose (Sigma-Aldrich, Burlington, MA, USA)	—	—	70 g	—
Urea (Sigma-Aldrich, Burlington, MA, USA)	—	—	0.64 g	—
NaCl (Sigma-Aldrich, Burlington, MA, USA)	—	—	1 g	—
(NH_4_)_2_SO_4_ (Sigma-Aldrich, Burlington, MA, USA)	—	—	1 g	—
MnSO_4_·7H_2_O (Sigma-Aldrich, Burlington, MA, USA)	—	—	—	1 g
CaCl_2_ (Sigma-Aldrich Massachusetts, USA)	—	—	—	2.3 g
CoCl_2_·6H_2_O (Sigma-Aldrich, Burlington, MA, USA)	—	—	—	2 g
FeSO_4_·7H_2_O (Sigma-Aldrich, Burlington, MA, USA)	—	—	—	3 g
ZnCl_2_ (Sigma-Aldrich, Burlington, MA, USA)	—	—	—	1 g
Traces solution	—	—	10 mL	—

### Appendix A.2

**Table A2:** Technical variability of OD600 measurements during the preliminary GCM growth-curve experiment. Values represent the mean ± standard deviation (SD) of three technical OD600 measurements obtained from the same batch culture at each sampling time.

Time (h)	OD600, Mean ± SD
0	0.002 ± 0.000
2	0.012 ± 0.000
4	0.234 ± 0.001
6	0.253 ± 0.006
24	0.575 ± 0.005
30	1.131 ± 0.013
48	1.291 ± 0.003
72	1.272 ± 0.001
96	1.076 ± 0.004

### Appendix A.3

**Table A3:** Temporal values of optical density, viable bacterial concentration, and aniline-blue reactivity in GCM and FCM. OD600 represents culture optical density measured at 600 nm; CFU/mL represents viable cultivable bacterial concentration; and A610 represents aniline-blue reactivity measured at 610 nm. These parameters were measured independently and are presented for descriptive temporal comparison. No quantitative equivalence among these measurements is implied.

Time (h)	GCM OD600	GCM CFU/mL	FCM OD600	FCM CFU/mL	FCM A610
0	0.016	4 × 10^5^	0.140	1.2 × 10^08^	0.240
2	0.002	4 × 10^5^	0.236	9.6 × 10^08^	0.353
4	0.016	4 × 10^5^	0.260	8 × 10^15^	0.541
6	0.028	1.2 ×10^6^	0.310	8.4 × 10^15^	0.962
24	0.006	3 × 10^7^	0.490	4.16 × 10^16^	0.964
30	0.158	8 × 10^7^	0.675	4.8 × 10^19^	1.042
48	1.102	9.6 × 10^7^	1.324	4 × 10^18^	1.053
72	2.306	2.44 ×10^21^	2.142	8 × 10^18^	1.057
96	2.434	1.08 × 10^21^	2.816	1.2 × 10^19^	1.058
